# Effectiveness of the Electronic Cigarette: An Eight-Week Flemish Study with Six-Month Follow-up on Smoking Reduction, Craving and Experienced Benefits and Complaints

**DOI:** 10.3390/ijerph111111220

**Published:** 2014-10-29

**Authors:** Karolien Adriaens, Dinska Van Gucht, Paul Declerck, Frank Baeyens

**Affiliations:** 1 Faculty of Psychology and Educational Sciences, KU Leuven—University of Leuven, Tiensestraat 102, 3000 Leuven, Belgium; E-Mails: karolien.adriaens@hotmail.com (K.A.); dinska.vangucht@thomasmore.be (D.V.G.); 2 Thomas More University College Antwerp, Molenstraat 8, 2018 Antwerp, Belgium; 3 Department of Pharmaceutical and Pharmacological Sciences, KU Leuven—University of Leuven, O&N II Herestraat 49, 3000 Leuven, Belgium; E-Mail: paul.declerck@pharm.kuleuven.be

**Keywords:** electronic cigarette, smoking reduction, tobacco harm reduction

## Abstract

*Background*: Smoking reduction remains a pivotal issue in public health policy, but quit rates obtained with traditional quit-smoking therapies remain disappointingly low. Tobacco Harm Reduction (THR), aiming at less harmful ways of consuming nicotine, may provide a more effective alternative. One promising candidate for THR are electronic cigarettes (e-cigs). The aim of this study was to investigate the efficacy of second-generation e-cigs both in terms of acute craving-reduction in the lab and in terms of smoking reduction and experienced benefits/complaints in an eight-month Randomized Controlled Trial (RCT). *Design*: RCT with three arms. *Methods*: Participants (*N* = 48) unwilling to quit smoking were randomized into two e-cig groups and one control group. During three lab sessions (over two months) participants, who had been abstinent for four hours, vaped/smoked for five minutes, after which we monitored the effect on craving and withdrawal symptoms. eCO and saliva cotinine levels were also measured. In between lab sessions, participants in the e-cig groups could use e-cigs or smoke *ad libitum*, whereas the control group could only smoke. After the lab sessions, the control group also received an e-cig. The RCT included several questionnaires, which repeatedly monitored the effect of *ad libitum* e-cig use on the use of tobacco cigarettes and the experienced benefits/complaints up to six months after the last lab session. *Results*: From the first lab session on, e-cig use after four hours of abstinence resulted in a reduction in cigarette craving which was of the same magnitude as when a cigarette was smoked, while eCO was unaffected. After two months, we observed that 34% of the e-cig groups had stopped smoking tobacco cigarettes, *versus* 0% of the control group (difference *p* < 0.01). After five months, the e-cig groups demonstrated a total quit-rate of 37%, whereas the control group showed a quit rate of 38% three months after initiating e-cig use. At the end of the eight-month study, 19% of the e-cig groups and 25% of the control group were totally abstinent from smoking, while an overall reduction of 60% in the number of cigarettes smoked per day was observed (compared to intake). eCO levels decreased, whereas cotinine levels were the same in all groups at each moment of measurement. Reported benefits far outweighed the reported complaints. *Conclusion*: In a series of controlled lab sessions with e-cig naïve tobacco smokers, second generation e-cigs were shown to be immediately and highly effective in reducing abstinence induced cigarette craving and withdrawal symptoms, while not resulting in increases in eCO. Remarkable (>50 pc) eight-month reductions in, or complete abstinence from tobacco smoking was achieved with the e-cig in almost half (44%) of the participants.

## 1. Introduction

According to the latest World Health Organization (WHO) Report on the Global Tobacco Epidemic [1], in 2011 the age-standardized estimated prevalence of smoking in Belgium among those aged 15 years or more was 27%. The most recent Belgian study [2] similarly shows that in 2013, 27% of Belgians aged 15–75 were current smokers, bringing Belgium close to the EU average smoking prevalence of 28% [3]. The main consequences of this smoking behavior are the many different pathophysiological effects resulting in cancers, cardiovascular and respiratory diseases, that eventually lead to high rates of premature death [3,4,5,6]. Importantly, many of the adverse pathophysiological effects, the years of life lost and the number of deaths caused by smoking can substantially be decreased when people reduce or stop smoking [4,6,7]. Despite all tobacco control efforts, however, reductions in smoking prevalence in Belgium—much like in the rest of Western Europe—appear to have stalled over the last decade [8].

Meta-analyses of Randomized Controlled Trials (RCT’s) show that traditional quit-smoking interventions are effective in comparison to placebo [9], but the six-month quit rates remain disappointingly low [10] and relapse rates are typically high [11]. Moreover, some recent studies have failed to find that these medications are effective in real-world use; e.g., the biologically validated smoking cessation rates for NRT and varenicline are typically around 10% or lower [12,13].

The fact that traditional quit-smoking interventions are largely unsuccessful for a majority of smokers, can probably be traced back to two implicit or explicit assumptions. A first is that smoking addiction equals nicotine addiction. It would be a crucial mistake to focus exclusively on nicotine and not on the many sensory and behavioral aspects of smoking that support both craving and smoking satisfaction [14,15]. A second implicit assumption of smoking cessation interventions is that the ultimate goal for all tobacco smokers trying to quit should be complete abstinence of nicotine and/or of any form of tobacco use. This goal may not be attainable, nor even desirable for many smokers, and tobacco harm reduction (THR) may offer a more feasible and realistic alternative in this respect. THR, working from the premise that “People smoke for nicotine but (…) die from the tar” [16,17], aims to reduce the health risks of tobacco smoking, by providing smokers with the opportunity to switch to (much) less harmful ways of consuming tobacco and/or nicotine [18,19,20,21]. This may include options like switching to smokeless tobacco (e.g., Swedish snus), long-term NRT, or more recently, the electronic cigarette (e-cig) [20,22,23]. The focus of this study is on the potential of the e-cig as a promising THR tool. While acknowledging the importance of efficient nicotine delivery, e-cigs also aim to address the behavioral and sensory aspects of smoking [23]. At the same time, a systematic review of current knowledge about the chemical composition, toxicological profile, and clinical safety of e-cigs indicates that they are several orders of magnitude less harmful than tobacco smoking, and probably also pose no more than minor health risks in an absolute sense [24,25,26]. This relative safety of e-cigs is largely due to the fact that they completely avoid the combustion of organic material (c.q., tobacco), and hence (most of the) toxic and carcinogenic chemicals that are present in cigarette smoke [27].

An e-cig consists of a battery, an electrical heating element (“atomizer”), and a replaceable or refillable cartridge with liquid (“e-liquid”) that contains propylene glycol and/or glycerol, water, food flavor(s), and (optionally) nicotine [27,28,29]. When activating the e-cig, the e-liquid is heated and transformed into a visible aerosol that can be inhaled and exhaled (“vapor”) by the user [29]; thus, delivering to the user not only nicotine [30], but also most of the critical sensory-motor cues associated with smoking (hand-to-mouth action, visual cues, throat hit, flavor). First-generation e-cigs are small cigarette look-alikes with low-capacity batteries and a heating element surrounded by a liquid-soaked poly-foam (“cartomizer”), whereas second-generation e-cigs typically have higher-capacity batteries, larger atomizers, and a refillable (transparent) tank setup (“clearomizer”), and typically produce more, thicker and also more consistent vapor [30].

The aim of this study was to investigate the efficacy of e-cigs both in terms of acute craving reduction in the lab and in terms of sustained smoking reduction, and to assess the experienced benefits and complaints in a RCT with three arms. The most important hypotheses were the following. First, we expected that in terms of acute craving for a tobacco cigarette the e-cig groups would show a significant decrease, but less so than the control group smoking a tobacco cigarette. We further hypothesized that the e-cig groups would show a learning curve, in that craving reduction was expected to gradually increase over lab sessions. Second, we expected that unlike in the control group smoking a tobacco cigarette, eCO levels in the e-cig groups would not increase immediately after using an e-cig; eCO baseline levels were also expected to decrease over sessions in the e-cig but not in the control groups. Third, the participants in the e-cig groups were expected to show a decrease in the number of cigarettes smoked per day during the first two months of the study, and more so than the control group participants. Finally, we hypothesized that the cotinine level in the e-cig groups would remain at the same level as in the control group.

## 2. Methods

### 2.1. General Layout of the Study

Participants with no intention to quit smoking were recruited and divided into three groups; two experimental/e-cig groups and one control group which kept on smoking regular tobacco cigarettes (during the first eight weeks of the study). In contrast to previous RCT’s, we made use of second-generation e-cigs because of the observation that regular vapers use these or even more advanced models [31] and because of the fact that these e-cigs perform better (more consistent vapor production and higher nicotine delivery) [30]. In addition, our study is one of the first to use a control group of smokers who were exposed to the same interventions and questionnaires as the experimental groups, thus allowing to control for the effects on smoking behavior of being involved in a study and of monitoring smoking behavior *per se*.

The first part of the study consisted of an eight-week lab study in which the previously discussed group division was maintained. In this part, participants were asked to come three times to the lab where we repeatedly examined the effect on craving and withdrawal symptoms of using an e-cig after being four hours abstinent from smoking or vaping. The choice to conduct three successive lab sessions was made to examine if there is any learning curve with respect to craving reduction when using an e-cig. Two objective physiological measurements (eCO and cotinine levels) were also included. In between these lab sessions, participants in the e-cig groups could use e-cigs or smoke *ad libitum*, whereas those in the control group could only smoke. This lab study was combined with a second part of the study that assessed the effect of using an e-cig *ad libitum* during several months on the use of tobacco cigarettes. The experienced benefits and complaints from using an e-cig or cigarette were also monitored by means of online questionnaires which participants were asked to fill out several times throughout the study. After the eight weeks of lab study, the control group became a “switch group”, in that participants could also use an e-cig from then on. This “switch group” allowed assessing any differences in smoking reduction and experienced benefits/complaints, between groups that were monitored and guided throughout their first weeks of using e-cigs (the experimental/e-cig groups), *versus* a group that was merely provided with an e-cig without any further guidance (the control/switch group). Finally, follow-up took place three and six months after the last lab session.

### 2.2. Participants

We recruited participants from the area around Leuven through various channels between December 2012 and February 2013. Following channels were used: (1) an advertisement on the official website of the KU Leuven Faculty of Psychology and Educational Sciences, (2) flyers on different campuses, (3) an internal e-mail to the personnel of the ATP (Administrative and Technical Personnel) group (people from the KU Leuven Kulak were also contacted through this channel), and (4) an advertisement in the local paper (Rondom Leuven). Inclusion criteria to participate in the study were the following: being a smoker for at least three years, smoking a minimum of 10 factory-made cigarettes per day and not having the intention to quit smoking in the near future, but willing to try out a less unhealthy alternative. Exclusion criteria included self-reported diabetes, severe allergies, asthma or other respiratory diseases, psychiatric problems, dependence on chemicals other than nicotine, pregnancy, breast feeding, high blood pressure, cardiovascular disease, currently using any kind of smoking cessation therapy and prior use of an e-cig. Participants decided through self-selection if they were eligible.

**Figure 1:**
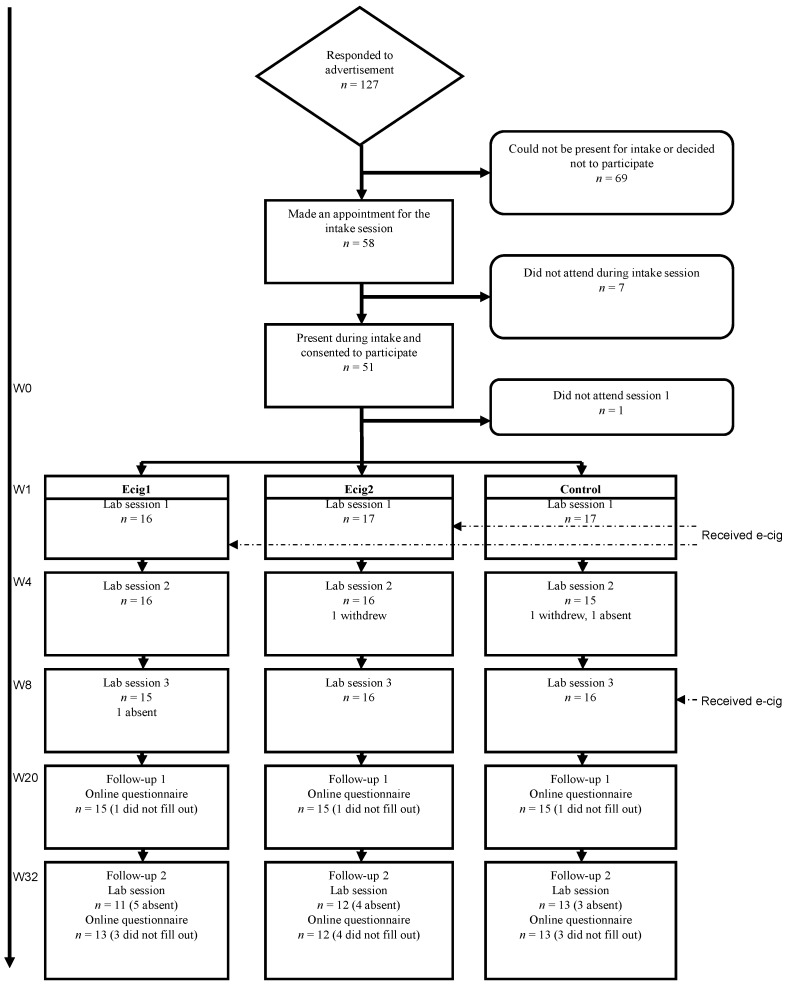
Participant flow.

We did not carry out a formal sample size calculation and used a convenience sample. A total of 127 people contacted us for more information about the study (see Figure 1). Eventually 48 participants completed the three lab sessions (three conditions of each 16 participants). During the first follow-up moment (FU1), three months after the last lab session, 45 participants filled out the questionnaire. During the second follow-up (FU2), six months after the last lab session, 38 participants completed the questionnaire and 36 participants were present during the final lab visit.

### 2.3. Materials

**Figure 2:**
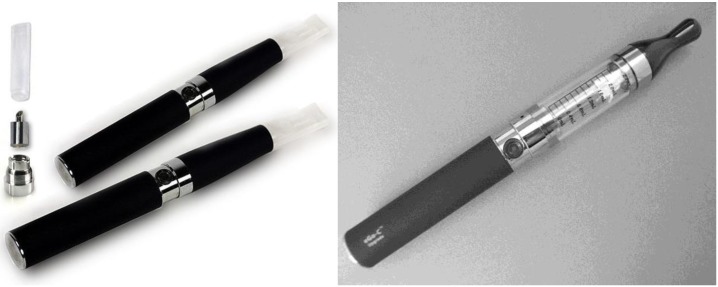
Materials.

Note: Joyetech eGO-C [35] on the left side and Kanger T2 CC [36] on the right side.

We used two different kinds of second generation e-cigs, namely the “Joyetech eGo-C” and the “Kanger T2-CC”, referred to as respectively type one and type two e-cigs. We chose to use two types of electronic cigarettes (See Figure 2) to compare potential model-related differences in e-cig efficacy, but we had no *a priori* hypotheses about the direction of eventual differences. The choice of these products was based on a subjective assessment of product-reviews on different e-cig forums.

The Joyetech eGo-C [32] consists of a rechargeable 1000 mAh 3.3 V lithium-ion battery, an atomizer body (cover cone and atomizer base) holding a refillable 1 mL cartridge serving as mouthpiece, and a replaceable 2.2-ohm atomizer head. The Kanger T2-CC [33] consists of a replaceable mouthpiece, a 2.4 mL clearomizer, a 2.5-ohm coil and a rechargeable 650 mAh 3.7 V lithium-ion battery. For both types of e-cigs we used 30 mL bottles of tobacco-flavored e-liquid (Dekang “Turkish Blend”), containing 18 mg/mL of nicotine [34]. Participants were encouraged to only use this type of e-liquid for reasons of standardization.

The e-cig groups received the e-cig and four bottles of e-liquid at Session 1 (group Ecig1 received the Joyetech eGo-C and group Ecig2 received the Kanger T2-CC); at Session 2, participants’ empty bottles were replenished up to again four bottles and at Session 3, they were allowed to keep the remaining bottles. For these groups, we performed multiple weightings, with a calibrated scale, of the 30 mL bottles containing the e-liquid to derive the average consumption of liquid per day in mL. The control group received the e-cig and e-liquid (six bottles) for two months at the end of Session 3 (eight of the 16 participants of the control group received the Joyetech eGo-C and the remaining eight participants received the Kanger T2-CC). All participants received their material for free. All devices and e-liquids for two months were provided by the experimenter.

### 2.4. Study Design and Procedures

The Medical Ethical Committee of the Faculty of Medicine, UZ Leuven, approved all of the measures and procedures used in this study.

Prior to the study, individuals could contact the first author for more information about the study. If they did so, they received a standardized e-mail with information about the general purpose and the course of the study. Those willing to participate were randomized to one of the three conditions: two e-cig conditions as experimental groups (Ecig1 and Ecig2) and one delay condition as a control group (Control). Block randomization was performed by using a randomization tool available on the website www.randomizer.org [37].

#### 2.4.1. Intake Session

We invited respondents in groups for an intake session of 90 min. In this timeframe, we told all participants to which condition they were assigned, provided general information on the e-cig, a current state of the art on the safety and relative risks of using e-cigs, a demonstration of the use of the products and a short explanation about the aim of the study. Afterwards, we scheduled their lab sessions. Apart from these practical arrangements, we asked participants to sign the informed consent and to fill out several questionnaires (see Section 2.5.2); finally, we conducted a breath carbon monoxide measurement (= baseline eCO).

At the end, we gave each participant an information pack with their dates for the lab sessions, a schedule on when to fill out the online diaries, four moments on which to collect saliva samples and finally one date for a final follow-up moment. We also added a bibliography of recent literature on the e-cig, a manual on the particular e-cig they would receive, a guide for novice vapers (http://hotcoildm.nl/hotcoildm-downloads/), instructions on how to collect the saliva samples, and contact details of the researchers. The actual e-cigs were not yet given to the participants during Intake. For the e-cig groups, the e-cig was given during Session 1 in which they had their first experience with it. At the end of that first session, those participants could take their e-cig home. The control group only received their e-cig at the end of Session 3.

#### 2.4.2. Laboratory Sessions

During the following eight-week period, participants were asked to come three times (Session 1 in week one, Session 2 in week four, Session 3 in week eight) to a lab session; each session lasted approximately one hour and was scheduled in the afternoon at either 2:00 p.m. or at 4:00 p.m. Participants were tested in groups with a minimum of two and a maximum of eight individuals of the same condition.

Participants were requested to abstain from smoking and vaping for at least four hours prior to each lab session. At the start of the session, participants took place in separate cubicles with the doors open and the experimenter collected the saliva samples (see Section 2.5.1) which participants had to self-provide the day before their scheduled lab session. Participants of the e-cig groups were also asked to bring their empty and full bottles of e-liquid to Session 2 and 3, so the used e-liquid could be measured.

First, participants filled out the questionnaires for the first time (T1, see Section 2.5.2). Then, we measured their eCO level (T1, see Section 2.5.1). This was followed by the instruction, for the e-cig groups, to take their provided e-cig and a short instruction on how to use the product. This instruction was only presented in the first session. Next, participants in the e-cig conditions were given five minutes to use the e-cig *ad libitum*. Participants in the control group were asked to go outside with the experimenter and were then given the opportunity to smoke their usual tobacco cigarette (or pipe) *ad libitum* during five minutes. We asked participants to fill out some ratings and to provide an eCO measurement immediately (T2), five (T3), 15 (T4), 30 (T5), and 50 (T6) minutes after the five minutes of smoking or vaping.

In the first (for the e-cig groups) and last (for the control group) sessions, the experimenter provided the e-cig and the predetermined quantity of e-liquid to the participants. In addition, information about the e-cig and about the potential purchase of the products was given. We, therefore, used the same website as initially used for the purchase of the products for this study (http://www.e-cig4u.nl/, see Section 2.3).

#### 2.4.3. Between Sessions

In addition to the lab sessions, we asked participants to fill out online questionnaires in the form of a diary (see Section 2.5.2). All questions were identical for all conditions, except no questions on the e-cig were included in the diaries of the control group. The diaries needed to be filled out on specific predetermined moments, namely on day one, three, five, seven, nine, 13, 17, 21, 25, 30, 35, 40, 45, 50, and 55. In order to make sure the time intervals were the same for all participants, the starting point (*i.e*., day one) coincided with the day of the first lab session.

#### 2.4.4. Follow-up

Three months after the last lab session (FU1), we asked all participants to fill out an online questionnaire assessing any changes in terms of smoking or vaping behavior (see Section 2.5.2). Six months after the last lab session (FU2), participants were invited to a follow-up session in which we provided some global information about the obtained preliminary results. We also collected final eCO data, saliva samples and we asked the participants to fill out some questionnaires (see Section 2.5.2).

### 2.5. Outcome Measures

#### 2.5.1. Physiological Measures

Throughout the study, we requested participants to collect four saliva samples, which were used to determine cotinine levels. There were several instructions to which participants needed to comply (e.g., no meal within the hour before collecting). The actual instructions for collecting the saliva consisted of placing a cotton swab behind the lower front teeth under the tongue for two minutes. Participants placed the used cotton swabs in a storage tube and then kept this tube in the refrigerator. We asked to bring the samples to each session and the final follow-up moment (FU2) and we stored them in a conventional freezer (−20 °C) before they were sent to the laboratory of Salimetrics who conducted the analyses by means of the Salimetrics Cotinine Salivary Immunoassay Kit [38].

During the lab sessions and the follow-up moment, we carried out multiple eCO measurements. The concentration (in ppm) of the exhaled breath CO was measured by using the piCO+ Smokerlyzer^®^ [39].

#### 2.5.2. Subjective Effect Questionnaires

During intake, participants filled out three questionnaires; 20 questions, assessing demographic variables and participants’ smoking history, the Fagerström Test for Cigarette Dependence (FTCD) [40] and the Beck Depression Inventory (BDI) [41].

During the lab sessions, we asked participants to fill out several questionnaires at multiple time points (see above). The control group was asked to fill out the Tobacco Craving Questionnaire (TCQ) [42], the Minnesota Nicotine Withdrawal Scale (MNWS) [43] and a visual analog scale (VAS) assessing cigarette craving. The TCQ consists of 12 items rated from 1 (totally disagree) to 5 (totally agree) [42]. This questionnaire measures craving for tobacco cigarettes. The MNWS consist of 15 items rated from 0 (not) to 4 (heavy) [43]. It is an assessment of nicotine withdrawal symptoms, such as feelings of anxiety or restlessness. The VASs were 100 mm, labeled on the left with ‘totally no craving’ and on the right with ‘very strong craving’. The same questionnaires were used for the two e-cig groups with the addition of a self-made e-cig version of the TCQ (same items as the original TCQ, but adapted to the e-cig) and a VAS concerning e-cig craving. Each lab session, the different measurements were repeated six times (T1 to T6, see above) except for the TCQ, which was only presented twice (immediately before and after five minutes of vaping/smoking).

In between the three lab sessions, we asked participants to fill out online diaries at 15 fixed time moments (see above). The control group received questions concerning current cigarette use, perceived complaints and benefits of smoking and mood, all with regards to the day preceding the online diary moment. For the e-cig groups the same questions were asked, but in relation to the e-cig. Additionally, additional questions about the e-cig were asked (e.g., current use, satisfaction and usability).

Finally, at FU1 and FU2, all participants were asked to again complete the online diary, which was the same as the one the e-cig groups filled out in between lab sessions. During the follow-up session (FU2), we also asked participants to fill out the BDI and a self-made questionnaire, which mainly assessed their current craving for a cigarette.

### 2.6. Statistical Analyzes 

For the data on participants’ characteristics we used descriptive analyses. The majority of our study outcome measures were analyzed by means of ANOVAs. For most variables measured during the lab sessions, including eCO, craving for a cigarette or e-cig and withdrawal symptoms, we carried out 3 (Group: Ecig1 *vs.* Ecig2 *vs.* Control) × 3 (Session: Session 1 *vs.* Session 2 *vs.* Session 3) × 6 (Time: T1 to T6) ANOVAs and subsequent planned comparisons with Group as between subjects variable and Session and Time as within-subjects variables. In a next step, these analyzes were expanded by adding FU1 and FU2. To compare the results of eCO at specific moments, 3 (Group: Ecig1 *vs.* Ecig2 *vs.* Control) × 2 (Moment: (for example: Intake *vs.* W1)) ANOVAs and subsequent planned comparisons were performed. Similar analyzes were carried out for the cotinine saliva data, namely 3 (Group: Ecig1 *vs.* Ecig2 *vs.* Control) × 3 (Session: Session 1 *vs.* Session 2 *vs.* Session 3) ANOVAs. Again, these analyzes were expanded in a next step by adding FU2.

Outcome measures obtained in the second part of the RCT, namely “Number of cigarettes smoked per day”, “Complaints”, and “Benefits”, were analyzed using similar 3 (Group: Ecig1 *vs.* Ecig2 *vs.* Control) × 6 (Moment: Intake *vs.* W1 *vs.* W2 *vs.* W3_4 *vs.* W5_6 *vs.* W7_8) ANOVAs and subsequent planned comparisons were performed with Group as between subjects variable and Moment as a within-subjects variable. To complete these analyzes, we added FU1 and FU2 to the comparisons. Finally, for the variables pertaining to e-cig use, 2 (Groups: Ecig1 *vs.* Ecig2) **×** 5 (Moment: W1 *vs.* W2 *vs.* W3_4 *vs.* W5_6 *vs.* W7_8) ANOVAs and subsequent planned comparisons were performed.

Reduction in smoking consumption from Intake to moments W7_8, FU1 and FU2 was assessed by classifying participants as failures (<50% reduction), 50% reduction (50%–80% reduction), 80% reduction (>80% reduction) and quitters (no more cigarette smoking). Participants with missing data were considered failures. The number of participants in each category was compared between groups (Ecig1/Ecig2 and controls) by chi-square tests.

## 3. Results

### 3.1. Participants’ Characteristics

Our total group consisted of 48 participants of whom 27 were female (see Table 1). The mean age was 43.71 years (*SD* = 13.13). At time of intake, they smoked on average 19.15 (*SD* = 7.41) cigarettes per day, and obtained an average score of 5.79 (*SD* = 1.70) on the FTCD, which indicates moderately strong cigarette dependence. The average eCO at baseline was 17.58 (*SD* = 7.17) ppm. There were no indications for the presence of depression (BDI-score *M* = 5.51, *SD* = 8.35).

The participant group consisted of people who on average had a slightly higher-than-average education level and were situated in an above-average income category. The majority of the participants worked full-time, some worked part-time or were students.

In terms of smoking history, most participants started smoking before the age of 20 (91.67%). On average, participants undertook 1.60 (*SD* = 2.03) quit attempts, mainly with the help of nicotine replacement therapy (25%) or willpower (37.50%). Overall, at intake, there were only two participants (4.17%) who had concrete plans to quit smoking. This was in line with the group we wanted to appeal.

There were no significant differences between the groups for any of these variables (all *p*s > 0.21).

**Table 1:** Participants’ characteristics.

Group	Gender	Age	% Employed	# Cigarettes	FTCD	BDI	eCO
Ecig1	7/9	44.75 (13.54)	78.75	20.13 (9.41)	5.81 (1.94)	6.81 (7.06)	19.13 (6.11)
Ecig2	10/6	46.06 (12.76)	71.25	20.63 (6.62)	6.31 (1.45)	6.14 (11.99)	17.38 (6.29)
Control	10/6	40.31 (13.21)	74.69	16.69 (5.49)	5.24 (1.62)	3.56 (4.34)	16.25 (8.92)
All groups	27/21	43.71 (13.13)	74.90	19.15 (7.41)	5.79 (1.70)	5.51 (8.35)	17.58 (7.17)

Note: all values means, except gender is a ratio female/male, *SD* between ( ); *n*_Ecig1_ = 16, *n*_Ecig2_ = 16, *n*_Control_ = 16, *n*_All groups_ = 48.

### 3.2. eCO Levels

**Figure 3:**
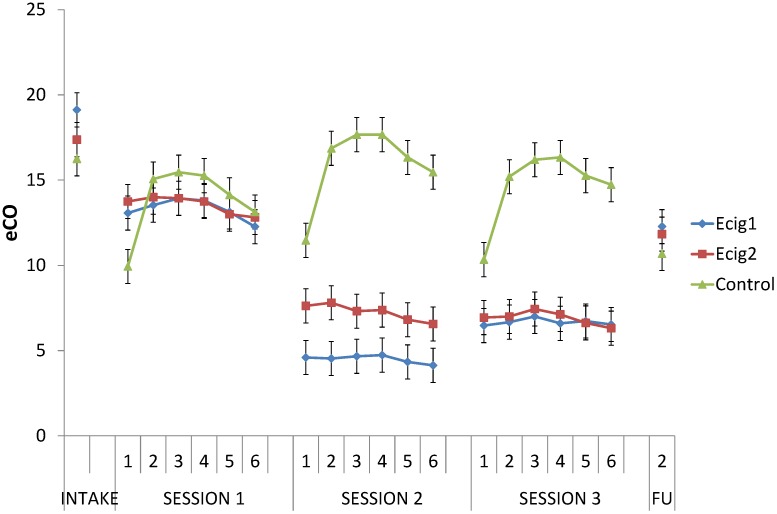
eCO measurements.

Note: all values mean (+/− 1 SEM) eCO levels; *n*_Ecig1—Intake_ = 16, *n*_Ecig1—Session 1/2/3_ = 15, *n*_Ecig1—FU2_ = 11; *n*_Ecig2—Intake_ = 16, *n*_Ecig2—Session 1/2/3_ = 16, *n*_Ecig2—FU2_ = 12; *n*_Control—Intake_ = 16, *n*_Control—Session 1/2/3_ = 15, *n*_Control—FU2_ = 13.

All groups combined showed a significant decrease, *F* (1, 45) = 42.56, *p* < 0.001, from Intake to the start of Session 1; with no difference between groups, *F* < 1 (see Figure 3). Average eCO decreased about 30%, from 17.58 ppm (*SD* = 7.17) at baseline to an average of 12.38 ppm (*SD* = 5.08) at the start of Session 1, indicating good compliance among participants with regards to the abstinence instruction.

Across sessions, the change in the level of eCO from T1 to T2 differed between groups, *F* (1, 43) = 124.57, *p* < 0.001; in the control group, the level increased from T1 to T2 (immediately after smoking), *F* (1, 43) = 198.87, *p* < 0.001; while, as expected, in the e-cig groups, no such increase in eCO level was observed from T1 to T2 (after vaping), *F* < 1. Across sessions, both e-cig groups had substantially lower eCO levels compared to the control group on T2, T3, T4, T5 and T6, all *p*s < 0.001. Importantly, only the e-cig groups, *F* (1, 43) = 78.23, *p* < 0.001, but not the control group, *F* (1, 43) = 2.88, *p* = 0.10, showed an overall decrease in eCO from Session 1 to Session 2; and this change reliably differed between groups, *F* (1, 43) = 41.54, *p* < 0.001. The same pattern could be observed when comparing the change in overall eCO levels of Session 1 to those of Session 3 (e-cig groups, *F* (1, 43) = 60.39, *p* < 0.001; control group, *F* < 1; interaction effect, *F* (1, 43) = 25.05, *p* < 0.001).

Finally, for all groups combined, there was a decrease in eCO level, *F* (1, 33) = 10.26, *p* < 0.01, from Intake to FU2; with no difference between groups, *F* < 1. Eventually, the average eCO at FU2 was 11.56 ppm (*SD* = 10.41).

For all the comparisons above, there were no differences between the two e-cig groups (all *p*s > 0.15).

### 3.3. Saliva Cotinine Levels

**Figure 4:**
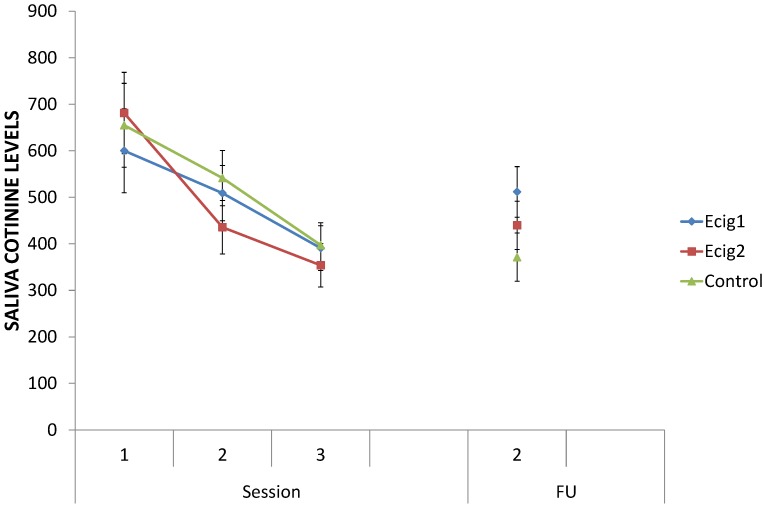
Saliva cotinine levels.

Note: all values mean (+/- 1 SEM) saliva cotinine levels; *n*_Ecig1—Session 1/2/3_ = 15, *n*_Ecig1—FU2_ = 11, *n*_Ecig2—Session 1/2/3_ = 16, *n*_Ecig2—FU2_ = 12, *n*_Control—Session 1/2/3_ = 15, *n*_Control—FU2_ = 12.

Irrespective of session (Session 1, 2 and 3), no differences could be found in the cotinine levels (ng/mL) between the e-cig groups and the control group, all *p*s > 0.34 (see Figure 4). For all groups combined, there was a decrease in cotinine levels from Session 1 to Session 2, *F* (1, 43) = 12.29, *p* < 0.01, and from Session 2 to Session 3, *F* (1, 43) = 15.30, *p* < 0.001. From each session to the next, there was no difference in the degree of the decrease in cotinine levels between the two e-cig groups and the control group, all *p*s > 0.48; the decrease was of the same magnitude for all groups. When including the final follow-up session (FU2), no difference between the e-cig groups and the control group could be obtained for FU2, *F* (1, 32) = 2.64, *p* = 0.11. From Session 3 to FU2, the two e-cig groups showed a little increase in cotinine level, *F* (1, 32) = 5.37, *p* < 0.05, while the control group did not show any increase, *F* < 1; the difference between the e-cig groups and the control group was significant, *F* (1, 32) = 4.07, *p* < 0.05. Despite this increase for the e-cig groups, there was a general decrease in cotinine level from Session 1 to FU2 for the e-cig groups, *F* (1, 32) = 9.37, *p* < 0.01, and for the control group, *F* (1, 32) = 16.01, *p* < 0.001, whereby the degree of the decrease was the same for all groups, *F* (1, 32) = 2.10, *p* = 0.16. The average cotinine level across all participants showed a decrease from 663.50 ng/mL (*SD* = 350.15) at baseline (Session 1) to 449.96 ng/mL (*SD* = 193.19) at the end of the study (FU2). For each of the analyses above, no differences between the two e-cig groups were significant, all *p*s > 0.15.

Second, one way ANOVA’s were carried out to check if there were any differences in the cotinine levels when we looked at subgroups of participants with different tobacco cigarette reduction rates (namely failure, ≥50% reduction, ≥80% reduction and quitters) for each specific moment. At each moment (Session 2, Session 3 and FU2) separately, no differences could be found between the categories of failure, 50% reduction, 80% reduction and quitters, all *p*s > 0.05 (see Table 2 for means). Only at the end of the study (FU2), the category of failure showed a higher cotinine level than the category of 80% reduction, *F* (1, 32) = 6.86, *p* < 0.05.

Finally, at Session 2, Session 3 and FU2 test moments separately, correlations were calculated between the number of tobacco cigarettes smoked by each participant and the level of cotinine. At each moment a weak positive relation between the number of cigarettes smoked and the cotinine level was obtained, all *r*s < 0.43. All correlations differed significantly from zero.

**Table 2:** Saliva cotinine levels per subgroup.

Reduction Rate	Session 2	Session 3	FU2
Failure	574.19	404.98	545.23
	(49.36)	(36.53)	(46.32)
	*n* = 22	*n* = 25	*n* = 15
≥50% reduction	368.16	435.03	356.49
	(94.52)	(69.04)	(89.71)
	*n* = 6	*n* = 7	*n* = 4
≥80% reduction	510.30	380.93	330.20
	(77.17)	(91.33)	(67.81)
	*n* = 9	*n* = 4	*n* = 7
Quitter	426.09	302.77	428.27
	(73.21)	(55.08)	(56.74)
	*n* = 10	*n* = 11	*n* = 10

Note: all values means, *SD* between ( ); *n* = number of participants.

### 3.4. Subjective Effect Questionnaires

#### 3.4.1. Craving for Cigarettes

**Figure 5:**
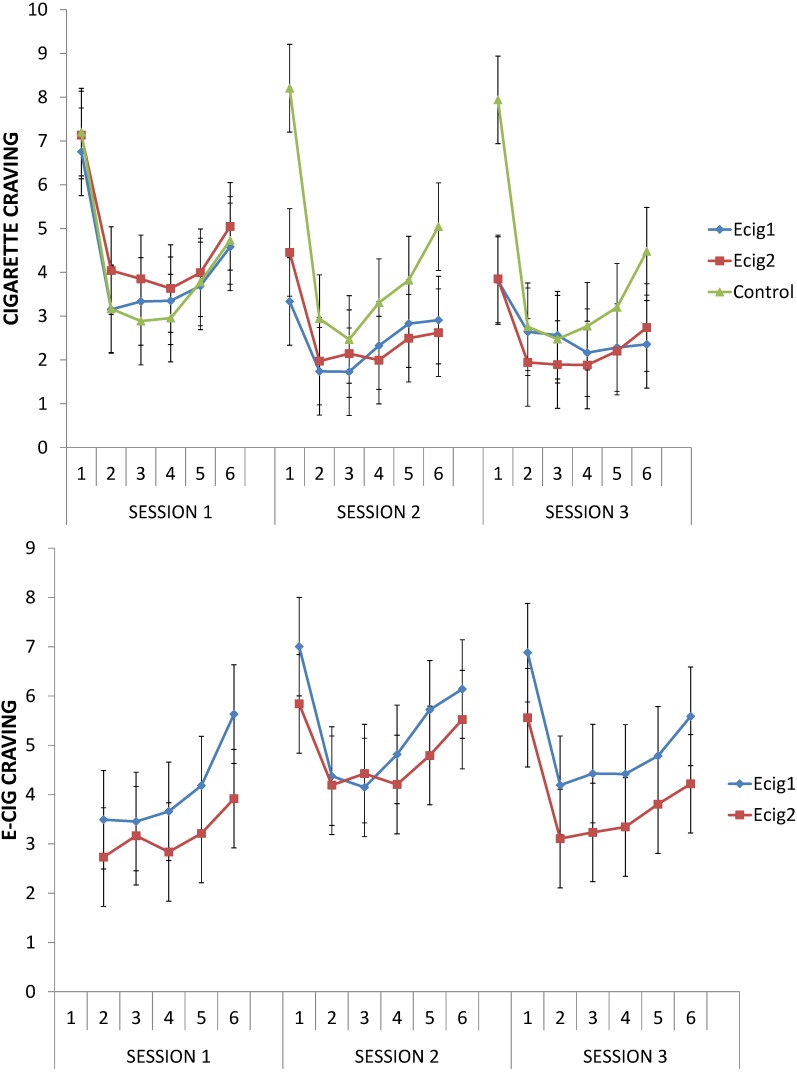
Cigarette and e-cig craving.

Note: top figure: all values mean (+/− 1 SEM) craving levels with minimum 0 and maximum 10; *n*_Ecig1—Session 1/2/3_ = 14, *n*_Ecig2—Session 1/2/3_ = 14, *n*_Control—Session 1/2/3_ = 15; bottom figure: all values mean (+/− 1 SEM) e-cig craving levels with minimum 0 and maximum 10; *n*_Ecig1—Session 1/2/3_ = 14, *n*_Ecig2—Session 1/2/3_ = 14. No measurement of e-cig craving at the start (T1) of Session 1, because participants had not yet used the e-cig.

At the start of Session 1, participants had on average a craving of 7.01 (*SD* = 2.52; *min.* = 0, *max.* = 10). After five minutes of smoking or vaping (T2), both e-cig groups, *F* (1, 40) = 39.22, *p* < 0.001, and the control group, *F* (1, 40) = 30.53, *p* < 0.001, showed a clear decrease in craving compared to the start of the session (T1); the degree of the decrease was the same for all groups, *F* < 1 (see Figure 5, top). Cigarette craving decreased to 3.28 (*SD* = 2.54). A decrease in craving from T1 to T2 was also observed in Session 2 (e-cig groups, *F* (1, 40) = 19.88, *p* < 0.001; control group, *F* (1, 40) = 70.94, *p* < 0.001) and Session 3 (e-cig groups, *F* (1, 40) = 33.16, *p* < 0.001, control group, *F* (1, 40) = 84.45, *p* < 0.001). For these sessions, however, the decrease for the e-cig groups was less pronounced than for the control group (interactions: Session 2, *F* (1, 40) = 17.33, *p* < 0.001; Session 3, *F* (1, 40) = 27.18, *p* < 0.001). This difference was also reflected in a significant 2 (Group: Ecig1/Ecig2 *vs.* Control) × 2 (Moment: Session 1, T1 *vs.* Session 2, T1) interaction, *F* (1, 40) = 23.46, *p* < 0.001, which was due to a between group-difference of craving levels at the start of both sessions, with significantly lower cigarette craving for the e-cig groups at the start (T1) of Session 2 than at the start (T1) of Session 1, *F* (1, 40) = 38.06, *p* < 0.001, but not for the control group, *F* (1, 40) = 2.21, *p* = 0.15. The same pattern could be observed from the start (T1) of Session 1 to the start of Session 3 (decrease in e-cig groups, *F* (1, 40) = 30.19, *p* < 0.001; no decrease in control group, *F* < 1; interaction, *F* (1, 40) = 16.10, *p* < 0.001).

An increase in craving could be observed from immediately after the five minutes of smoking or vaping (T2) to the end (T6) of Session 1 and 2 for both the e-cig groups (Session 1, *F* (1, 40) = 8.24, *p* < 0.01; Session 2, *F* (1, 40) = 4.19, *p* < 0.05) and the control group (Session 1, *F* (1, 40) = 7.23, *p* < 0.05; Session 2, *F* (1, 40) = 12.02, *p* < 0.01); the degree of the increase was the same for all groups (Session 1, *F* < 1; Session 2, *F* (1, 40) = 2.52, *p* = 0.12). At Session 3, the change in craving from T2 to T6 differed between groups, *F* (1, 40) = 7.44, *p* < 0.01; in the control group, there was an increase in the craving for cigarettes, *F* (1, 40) = 15.71, *p* < 0.001; while in the e-cig groups, no change in cigarette craving was observed, *F* < 1.

Overall, there were no significant differences for any of the reported effects between the two e-cig groups (all *p*s > 0.09). These results were confirmed by the scores on the TCQ.

#### 3.4.2. Craving for e-Cigs

For Sessions 2 and 3, there was a significant decrease in e-cig craving from the start of the session (T1) to immediately after vaping (T2) for both e-cig groups, all *p*s < 0.001 (see Figure 5, bottom). During every session, the two e-cig groups showed an increase from immediately after vaping (T2) to the end of the session (T6), all *p*s < 0.01. Overall, the two e-cig groups did not differ from each other, all *p*s > 0.20. These results were also confirmed by the results of the e-cig variant of the TCQ.

#### 3.4.3. Withdrawal Symptoms

In all sessions, across all groups, a decrease in withdrawal symptoms from the start of the session (T1) to immediately after the five minutes of smoking or vaping (T2) was present, all *p*s < 0.001; and MNWS scores stayed at the same level from immediately after the five minutes of smoking or vaping (T2) to the end of all sessions (T6), all *p*s > 0.05.

#### 3.4.4. Number of Cigarettes per Day

In order to minimize the impact of missing data, we reduced the 15 data points for each participant (see above) to averages at five periods defined in terms of the weeks corresponding to the eight weeks of the lab study (W1, W2, W3_4, W5_6, W7_8). Each period consists of an average of three consecutive data points; thus, for example, W1 is the average of the three first data points. Only for W7_8 an average of two data points was used, because the very last data point was beyond the duration of the lab study and the control group already had been offered their e-cigs at that moment.

**Figure 6:**
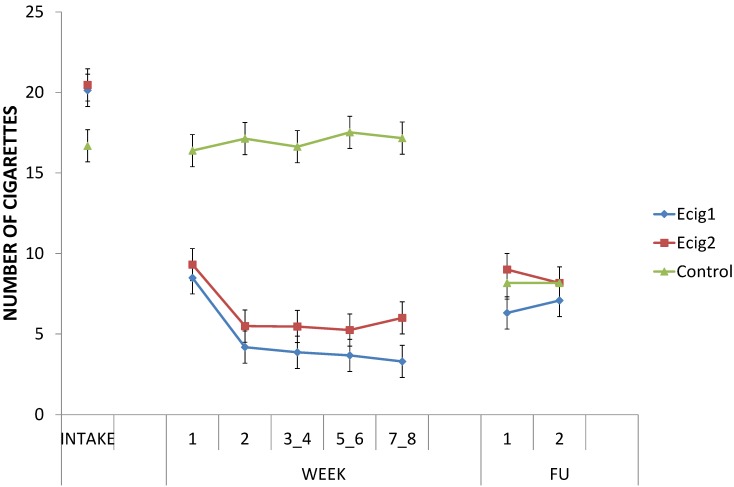
Number of cigarettes/day.

Note: all values mean (+/− 1 SEM) number of cigarettes; *n*_Ecig1—Intake_ = 15, *n*_Ecig1—W1–W7_8_ = 12, *n*_Ecig1—FU1_ = 13, *n*_Ecig1—FU2_ = 13; *n*_Ecig2—Intake_ = 15, *n*_Ecig2—W1–W7_8_ = 13, *n*_Ecig2—FU1_ = 12, *n*_Ecig2—FU2_ = 12; *n*_Control—Intake_ = 16, *n*_Control—W1–W7_8_ = 15, *n*_Control—FU1_ = 12, *n*_Control—FU2_ = 12.

First, the change in the number of cigarettes smoked per day from Intake to the first week (W1) differed between groups, *F* (1, 37) = 28.21, *p* < 0.001; the e-cig groups immediately showed a substantial reduction in the number of cigarettes smoked, *F* (1, 37) = 86.04, *p* < 0.001, while this amount remained stable in the control group, *F* < 1 (see Figure 6). In addition, from W1 to W2 also a reduction in the number of cigarettes for the e-cig groups was present, *F* (1, 37) = 42.76, *p* < 0.001. At each of the weeks as well as averaged over all weeks of the lab study (W1, W2, W3_4, W5_6, W7_8), the e-cig groups smoked substantially less cigarettes, respectively all *p*s < 0.001 and (1, 37) = 36.69, *p* < 0.001.

The control group had received an e-cig during the last lab session (after W7_W8), and, thus, had the opportunity to experiment with it for three months at FU1. The difference in the number of cigarettes smoked that was present between the groups throughout the lab study (W1 to W7_8), was no longer present when we looked at the change from Intake to the first follow-up (FU1) measurement, *F* (1, 35) = 1.27, *p* = 0.27; both the control group, *F* (1, 35) = 11.77, *p* < 0.01, and the e-cig groups, *F* (1, 35) = 44.62, *p* < 0.001, showed a decrease in number of cigarettes smoked from Intake to FU1; the degree of the decrease was the same for all groups, *F* (1, 35) = 1.27, *p* = 0.27. At FU1, the groups smoked the same amount of cigarettes, *F* < 1.

The pattern in the change of the number of cigarettes that was present from Intake to FU1 (see above), could also be observed from Intake to FU2; both the control group, *F* (1, 32) = 14.29, *p* < 0.001, and the e-cig groups, *F* (1, 32) = 43.27, *p* < 0.001, showed a decrease in number of cigarettes smoked from Intake to FU2; the degree of the decrease was the same for all groups, *F* < 1. Again, there was no difference between the groups in the number of cigarettes used at FU2, *F* < 1.

Finally, the overall average of the number of cigarettes smoked per day for the e-cig groups, decreased about 77% from 20.38 cigarettes (*SD* = 8.01) at baseline (Intake) to an average of 4.7 cigarettes (*SD* = 6.5) at the end of the lab study (W7_8). For all groups combined, a decrease of 51% was present from 19.15 cigarettes (*SD* = 7.41) at intake to an average of 9.38 cigarettes (*SD* = 8.58) at W7_8. Across all participants, a decrease of 60% in the number of cigarettes was obtained from baseline to FU2 (*M* = 7.66, *SD* = 7.72).

For all the analyses above, no differences between the two e-cig groups were found, all *p*s > 0.36.

**Figure 7:**
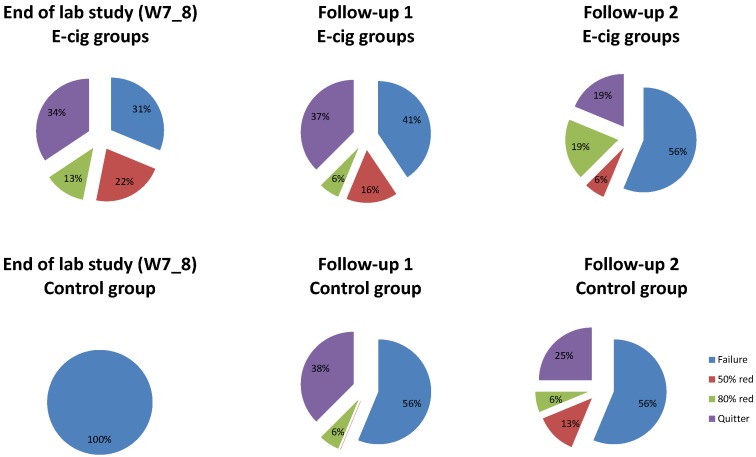
Reduction rates.

Note: all values reduction rates (%); *n*_Ecig groups—W7_8/FU1/FU2_ = 32, *n*_Control—W7_8/FU1/FU2_ = 16. The category “Failures” included 3%, 6%, and 28% of participants with missing data at W7_8, Follow-up 1, and Follow-up 2 in the E-cig groups, *versus* 0%, 6%, and 19% in the Control group. For quitters, the difference between e-cig groups and control group was statistically significant at W7_8 (p < 0.01).

For each participant, we calculated reduction rates (%) by comparing the self-reported number of cigarettes for some moments (W7_8, FU1, FU2) with the number of cigarettes used at Intake (see Figure 7). At the end of the lab study (W7_8), the distribution of the reduction rates differed between groups, χ^2^ (3) = 20.31, *p* < 0.001 (Yates χ ^2^ (3) = 15.13, *p* < 0.01). This difference disappeared at FU1, χ^2^ (3) = 3.07, *p* = .38 (Yates χ^2^ (3) = 1.94, *p* = 0.58), and at FU2, χ^2^ (3) = 1.84, *p* = 0.61 (Yates χ^2^ (3) = 0.53, *p* = 0.91). The high number of “quitters” at FU1, decreased at FU2 for both e-cig groups and the control group, but still 21% of all participants had completely stopped smoking (quitter) six months after the last lab session. The category of “quitter” was biologically verified with eCO levels; participants needed to show an eCO of 5 ppm or smaller.

#### 3.4.5. Complaints and Benefits

The various complaints and benefits of the cigarette or e-cig questioned in the online diaries are described in Table 3. Separately for the complaints and benefits a total score (which only included the complaints and benefits of the upper section of Table 3), ranging from 1 to 10, was calculated by taking the average of all the complaints or benefits at each moment (W1 *vs.* W2 *vs.* W3_4 *vs.* W5_6 *vs.* W7_8 *vs.* FU1 *vs.* FU2) separately.

**Table 3:** Complaints and benefits of the cigarette or e-cig.

Item relevant for	Complaints	Benefits
**Cigarette and e-cig**	Bad taste	Pleasant sensation when inhaling
	Dry mouth / throat	Improved breathing
	Irritated mouth / throat	Pleasant taste when inhaling
	Dizziness	Less coughing or sore throat
	Headache	Improved health and fitness
	Nausea	Helps to reduce or stop smoking
	Increased heart rate/palpitations	Improved taste and smell
	Increased weight	Less unpleasant smells
	Concerns about health risks	Improved sleep
**E-cig**	Technical problems	Pleasure of vaping
		Less desire for cigarettes
		Fresher breath
		Can be used in more places
		I bother others less with the e-cig

**Figure 8:**
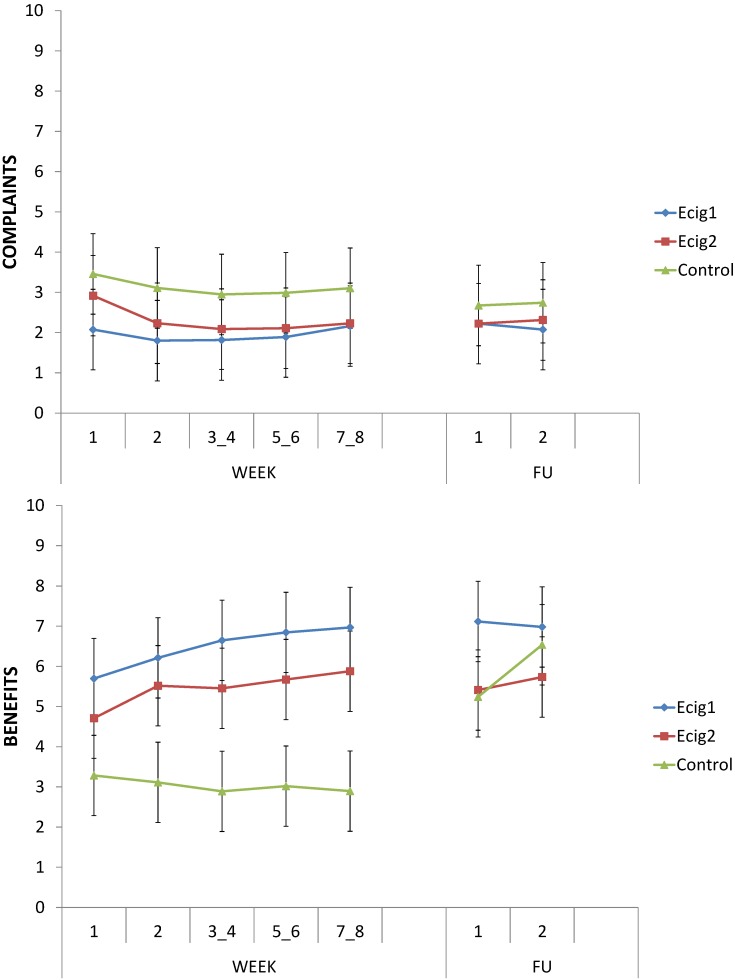
Complaints and Benefits.

*Note*: top figure: all values mean (+/− 1 SEM) total score complaints with minimum 0 and maximum 10; *n*_Ecig1—W1-W7_8_ = 12, *n*_Ecig1—FU1_ = 12, *n*_Ecig1—FU2_ = 12; *n*_Ecig2—W1–W7_8_ = 13, *n*_Ecig2—FU1_ = 12, *n*_Ecig2—FU2_ = 12; *n*_Control—W1–W7_8_ = 15, *n*_Control—FU1_ = 13, *n*_Control—FU2_ = 13; bottom figure: all values mean (+/− 1 SEM) total score benefits with minimum 0 and maximum 10; *n*_Ecig1—W1–W7_8_ = 12, *n*_Ecig1—FU1_ = 13, *n*_Ecig1—FU2_ = 13; *n*_Ecig2—W1–W7_8_ = 13, *n*_Ecig2—FU1_ = 10, *n*_Ecig2–FU2_ = 10; *n*_Control—W1–W7_8_ = 15, *n*_Control—FU1_ = 12, *n*_Control—FU2_ = 12.

As can be seen in Figure 8 (top), the level of complaints reported was low. Across all moments (W1 to W7_8), the control group reported more complaints about smoking cigarettes than the e-cig groups about using the e-cig, *F* (1, 37) = 7.30, *p* < 0.05. From the beginning (W1) to the end (W7_8) of the lab study, both the e-cig groups, *F* (1, 37) = 1.63, *p* = 0.21, and the control group, *F* (1, 37) = 1.37, *p* = 0.25, did not show any change in experienced complaints of the e-cig or cigarette, and the groups did not differ in this respect, *F* < 1.

At FU1 and FU2 the control group—having then switched to e-cigs—did no longer differ from the e-cig groups, *F* (1, 34) = 1.03, *p* = 0.32. Both the control group, *F* (1, 34) = 2.31, *p* = 0.14, and the e-cig groups, *F* < 1, did not show any change in experienced complaints from the start (W1) of the lab study to FU1, however.

In line with the previous, across all moments (W1 to W7_8), the e-cig groups were much more positive about the e-cig than the control group was about the cigarette, *F* (1, 37) = 47.34, *p* < 0.001 (see Figure 8, bottom). From the start (W1) to the end (W7_8) of the lab sessions, the groups differed in the change in perceived benefits, *F* (1, 37) = 14.48, *p* < 0.001; the e-cig groups showed an increase in experiencing benefits, *F* (1, 37) = 22.15, *p* < 0.001, while the control group did not show any change, *F* (1, 37) = 1.36, *p* = 0.25. From the start (W1) of the lab study to FU1, both the control group, *F* (1, 31) = 9.34, *p* < 0.01, and the e-cig groups, *F* (1, 31) = 4.27, *p* < 0.05, showed an increase in experienced benefits. Importantly, at FU1 the control group was using the e-cig. Across FU1 and FU2, both the e-cig groups and the control group reported many experienced benefits from the e-cig, with no differences between the groups, *F* < 1. No differences between the e-cig groups were observed for the reported benefits, all *p*’s > 0.07.

Finally, we also included benefits specific to the e-cig (see lower section of Table 3). Only a slight increase from the start (W1) to the end (W7_8) of the lab study for both e-cig groups was obtained, all *p*s < 0.05, for the following variables: “pleasure of vaping”, the fact that it “can be used in more places” and that “others will be bothered less” when using the e-cig. The other variables did not show any change in time, all *p*s > 0.13. For these benefits specific to the e-cig, there were no differences between the e-cig groups, all *p*s > 0.15, except for the aspect “others will be bothered less”, which group Ecig2 experienced more than group Ecig1, *F* (1, 23) = 4.32, *p* < 0.05.

#### 3.4.6. E-cig Use

Different aspects of e-cig use can be found in Table 4. For all variables, separate 2 (Group: Ecig1 *vs.* Ecig2) **×** 5 (Moment: W1 *vs.* W2 *vs.* W3_4 *vs.* W5_6 *vs.* W7_8) ANOVAs and subsequent planned comparisons with Group as between subjects variable and Moment as within-subjects variable were carried out. Additionally, for some variables FU1 and FU2 were added in a 2 (Group: Ecig1 *vs.* Ecig2) **×** 3 (Moment: W7_8 *vs.* FU1 *vs.* FU2) ANOVA. We mainly looked at the change from the start (W1) to the end (W7_8) of the lab study and to three (FU1) and six (FU2) months after the lab sessions.

From the start (W1) to the end (W7_8) of the lab study, an increase in the self-reported number of inhalations per day was present for both e-cig groups combined, *F* (1, 21) = 20.49, *p* < 0.001, followed by a non-significant decrease from (W7_8) to FU1 and FU2 (both *p*s > 0.06). Across all moments (W1 to W7_8) the two e-cig groups did not differ from each other *F* < 1.

**Table 4:** Aspects of e-cig use.

Group	# Inhalations Per Day				Liquid			
	W1	W7_8	FU1	FU2	W3_4		W7_8	
Ecig1	70.36	151.76	122.82	111.42	1.69		2.73	
	(70.75)	(126.58)	(117.27)	(105.34)	(0.98)		(1.74)	
	*n* = 15	*n* = 11	*n* = 14	*n* = 12	*n* = 16		*n* = 14	
Ecig2	66.64	85.25	60.86	46.36	1.07		1.49	
	(73.50)	(63.60)	(91.56)	(77.11)	(0.78)		(1.06)	
	*n* = 15	*n* = 13	*n* = 14	*n* = 11	*n* = 16		*n* = 16	
	**Technical Problems**				**Satisfaction**			
	**W1**	**W7_8**	**FU1**	**FU2**	**W1**	**W7_8**	**FU1**	**FU2**
Ecig1	2.74	1.82	2.07	2.75	7.24	8.08	5.80	6.46
	(2.87)	(1.27)	(1.21)	(2.14)	(1.30)	(1.73)	(3.36)	(3.13)
	*n* = 14	*n* = 11	*n* = 14	*n* = 12	*n* = 14	*n* = 12	*n* = 15	*n* = 13
Ecig2	2.48	3.65	4.00	4.92	6.89	7.54	6.73	6.36
	(2.23)	(3.23)	(3.44)	(3.65)	(1.41)	(1.99)	(2.60)	(3.11)
	*n* = 14	*n* = 13	*n* = 15	*n* = 12	*n* = 15	*n* = 13	*n* = 15	*n* = 11

Note: all values means with minimum 0 and maximum 10, except # inhalations per day is the average number of inhalations per day and liquid is the average ml used per day, SD between ( ).

On average, the first e-cig group was using more e-liquid (in ml/day) than the second e-cig group, *F* (1, 28) = 7.13, *p* < 0.05. From the first half (W3_4) to the second half (W7_8) of the lab study, an increase in e-liquid use in both groups was present, *F* (1, 28) = 11.00, *p* < 0.01.

In general, the ratings for experienced technical problems were low. Across all weeks of the lab study (W1 to W7_8), these ratings were different between the two e-cig groups, *F* (1, 21) = 4.62, *p* < 0.05, the second e-cig group was experiencing more technical problems than the first e-cig group. This observation was also present at FU1 and FU2, *F* (1, 19) = 5.99, *p* < 0.05.

Reported satisfaction with the e-cig was moderate to high and did not reliably differ between moments or groups, all *p*s > 0.10, except for the fact that the first e-cig group showed a decrease in satisfaction from the end (W7_8) of the lab study to FU1, *F* (1, 20) = 9.80, *p* < 0.01, and to FU2, *F* (1, 20) = 5.59, *p* < 0.05.

#### 3.4.7. Mood

At baseline, an average BDI-score of 5.79 (*SD* = 8.35) was obtained. This score did not differ much from the average score at FU2, which amounted to 4.94 (*SD* = 8.76), *F* < 1. In general, there were no indications for the presence of depressions during the whole study.

## 4. Discussion

Over the past years, the e-cig has become more popular; also awareness and use of the e-cig has shown a remarkable increase [44]. For the e-cig to be a useful THR tool, it is important to demonstrate its effectiveness with respect to craving reduction, smoking reduction, and experienced benefits and complaints. These aspects were the focus of this research in which we recruited participants who had no intention to quit smoking but were willing to try out a less harmful alternative. Importantly, and unlike in most previous studies [45,46,47,48], we used second-generation e-cigs, which are known to be used more often by daily e-cig users [31,49,50] and which appear to be more effective in nicotine delivery than first-generation e-cigs [31]. In addition, we worked with a control group that continued regular tobacco smoking during the lab study phase. This control group only received the e-cig kit after these eight weeks, which allowed us to assess the influence of guided *versus* non-guided switching to e-cigs.

The first part of this research included a lab study in which we repeatedly examined craving and withdrawal symptoms. In all three sessions and for all groups, strong reductions in cigarette craving were obtained after vaping or smoking for five minutes after having been abstinent for four hours. Even when using the e-cig for the very first time during Session 1 (e-cig groups), the decrease in cigarette craving was of the same magnitude as for the control group who smoked a tobacco cigarette. This was something we did not expect [51,52,53,54,55], because we assumed that some learning is needed to get used to an e-cig [56]. Previous studies on the efficacy and effectiveness of the e-cig already showed that the e-cig is able to suppress the desire to smoke to some extent: significant craving reductions have been observed, but typically less than after smoking a tobacco cigarette [51,52,53,54,55,57]. Our study demonstrates that an immediate and strong craving reduction by means of an e-cig is also possible. Two explanations could be offered for this effect: first, we used second-generation e-cigs (see above) and second, we gave clear instructions on the optimal use of an e-cig.

Another remarkable and hitherto not reported finding was that the e-cig groups also showed a lower cigarette craving after abstinence for four hours at Sessions 2 and 3 than at Session 1. Apparently, these participants’ cigarette craving had shifted to craving an e-cig. Finally, little withdrawal symptoms were reported, and these symptoms still decreased when the e-cig was used after being abstinent. This again is in contrast with previous research, where using an e-cig was also shown to reduce withdrawal symptoms, but less than smoking a tobacco cigarette [51,52,53,54,55,57].

The second part investigated the effect of being provided with an e-cig on smoking behavior, and assessed experienced benefits and complaints when using an e-cig over a period of eight months. All participants together showed a 60% decrease in the number of cigarettes used from intake to follow-up six months after the last lab session. Thus, a clear reduction in the number of cigarettes smoked per day was present when participants with no intention to quit smoking were given the opportunity to use an e-cig. This is consistent with results from survey research showing that when people buy or are offered an e-cig for the first time, they are likely to show a substantial reduction (or total abstinence) in the number of cigarettes smoked after six months [58,59]. Even when the e-cig could be used for no more than just one week *ad libitum*, it has been found that readiness and confidence to quit smoking can increase [60]. Similarly, in several well-documented case reports persons who repeatedly failed to quit smoking by means of classic smoking cessation methods, were able to quit smoking by means of the use of an e-cig [61,62].

A quit rate of 34% was obtained for the e-cig groups at the end of the lab study. There were no significant changes in smoking behavior in the control group over the first two months of the study. Hence, at least in this study, merely monitoring one’s own cigarette smoking behavior did not have any influence on reducing smoking in participants with no intention to quit. When we looked at the reduction rates three months after the last lab session (five months after the start of the study), 38% of all participants showed sustained complete abstinence from smoking (that is, 37% of the e-cig groups, and 38% of the control group (three months after initiating e-cig use)), 6% showed a reduction of more than 80%, another 10% showed a reduction of more than 50%, whereas the remaining 46% were smoking 50% or more of their number of cigarettes at baseline (including participants with missing data). At FU2 (six months after the last lab session and thus eight months after the start of the study), 21% of all participants showed sustained complete abstinence from smoking (that is, 19% of the e-cig groups and 25% of the control group (six months after initiating e-cig use)), 15% showed a reduction of more than 80%, another 8% showed a reduction of more than 50%, whereas the remaining 56% were smoking 50% or more of their number of cigarettes at baseline (including missing data). It seems that participants who decided to switch to an e-cig were relatively able to maintain this switch.

The quit rates obtained in this study are remarkable, especially in the light of the results of two earlier RCTs investigating the effectiveness of e-cigs. In one recent RCT, a comparison was made between the efficacy of e-cigs and nicotine patches for smoking cessation in participants wanting to quit smoking [63,64]. After six months, 7.3% of the participants were completely abstinent from tobacco cigarettes with nicotine e-cigs, 5.8% with nicotine patches and 4.1% with placebo (non-nicotine) e-cigs [63]; the differences between the groups were not statistically significant, however. In a second RCT, smokers not intending to quit were either offered nicotine-containing e-cigs or no-nicotine e-cigs for 12 weeks [46]. At week 12 complete abstinence from tobacco smoking was documented in 14.0% of the participants in groups with nicotine-containing e-cigs *versus* in 4.0% in the no-nicotine group, whereas at week 52 quit rates were 11.0% *versus* 4.0%, resp. Finally, in a series of prospective trials (without control group) of participants not intending to quit, quit rates were 14.5% to 22.5% depending on the duration of the follow-up, whereas a tobacco smoking reduction of at least 50% (including quitters) was achieved in 40%–55% of all participants [45,47,48]. Importantly, and unlike in the current study using second-generation e-cigs, these prospective studies and RCT’s used inefficient and now-obsolete first-generation e-cigs [45,46,47,48]. Hence, the modest quit rates in these earlier prospective trials and RCT’s can possibly be explained by the type of e-cig used. This explanation would be in line with what is known from survey research, in which the majority of regular vapers also reports to use second-generation e-cigs [28,31,49,50,65].

To combine the results of the second part of study with the lab study, we biologically confirmed the abstinence rates with exhaled breath CO. Using an e-cig for five minutes did not show any increase in eCO in contrast to when a tobacco cigarette was smoked. In Sessions 2 and 3 the e-cig groups showed a much lower average eCO than the control group did. This confirmed the self-reported decreases in the number of cigarettes smoked per day. The control group also showed a decrease in eCO at six months follow-up, confirming their switch to the e-cig. Most of the prospective studies and RCT’s described above also biologically verified self-reported abstinence or reduction by measuring levels of eCO. In line with our results, decreases in self-reported numbers of cigarettes smoked were observed to go hand in hand with decreases in eCO [45,46,48] or, in one study, in arterial and venous carboxyhaemoglobin [66].

With respect to the efficacy of nicotine delivery, a number of experiments investigated the e-cigs’ potential in participants that had been abstinent for a number of hours before using an e-cig for a limited number of puffs. Most of these studies used first-generation e-cigs, which were presented to e-cig-naïve tobacco smokers, and showed modest but significant increases in plasma nicotine levels [51,52,54,67,68], although smoking a tobacco cigarette typically showed a faster and stronger peak in blood nicotine concentration [30,51]. These generally weak-to-modest effects can possibly in part be explained by the lack of experience of the participants with e-cigs [67], in part by the fact that most experiments used e-cigs that were not good at delivering nicotine. In studies with more experienced e-cig users, a significant and stronger increase in plasma nicotine was obtained within five or 10 min after the first puff [52,68], and second-generation e-cigs were shown to deliver significantly more nicotine to the blood than first-generation e-cigs [30]. In contrast with these earlier lab results, our data show that saliva cotinine levels at the time of the lab sessions did not show any difference between experimental conditions: at each moment participants who quit smoking completely and switched to e-cigs as well as participants who used the e-cig in addition to some tobacco cigarettes (mixed use), showed the same cotinine levels as the control group that was still smoking tobacco cigarettes exclusively at that time. Likewise, at FU2 no between-group differences could be detected between the average cotinine levels. This suggests that the e-cigs used in this study were able to deliver nicotine efficiently, and that with some practice (exclusive or mixed) e-cig users adequately self-titrated nicotine intake. This finding is in line with observational studies showing that saliva cotinine levels can be of the same magnitude in experienced e-cig users as in smokers [67,69].

In prospective studies, RCT’s, internet surveys, case reports and interviews with e-cig users, the most frequently reported adverse effects when using an e-cig included headaches, dry mouth or throat, throat or mouth irritation, dry cough, nausea and technical problems; these mostly innocent side-effects were repeatedly shown to largely disappear during the course of the study, and side effects or withdrawal symptoms typically reported with classic smoking cessation tools were not or only infrequently reported [28,29,45,46,47,48,49,60,65,70]. Remarkably, in our research the control group reported more complaints on their tobacco cigarette than the e-cig groups did on their e-cig. This difference disappeared at FU1 and FU2, possibly due to the fact that the control group switched to the e-cig. Thus, the several adverse effects that have previously been described in studies not using a control group of smokers, probably overestimated the “e-cig-specific” adverse effects, because participants who continued smoking regular tobacco cigarettes were found to report more complaints than participants who used the e-cig. In addition, participants in the e-cig groups reported more experienced benefits from the e-cig than the control group experienced from tobacco cigarettes. Here we observed a possible learning effect, in that the e-cig groups showed an increase in experienced benefits when progressing through the study. After the lab study, the control group also showed an increase in experienced benefits when switching to the e-cig. As said earlier, no such learning effect was found for craving reduction: Participants who had never used an e-cig before showed an immediate strong decrease in cigarette craving.

A final objective of our study was to explore the influence of guided (e-cig groups) *versus* non-guided (control group) switching to e-cigs, the latter resembling real life e-cig use. The e-cig groups were monitored and guided through their first eight weeks of using e-cigs and the control group was merely provided with an e-cig without further guidance at the end of the lab sessions. We observed that the control group showed effects similar to the e-cig groups in terms of smoking reduction and experienced complaints/benefits when they switched on their own to the e-cig. This suggests that people who want to start with e-cigs on their own, may attain similar successes as those receiving substantial guidance; which is in accordance with results from survey research [50].

Besides these promising results in favor for the e-cig, our study has some limitations. First of all, our participants were slightly higher-than average educated. Second, our total group consisted of a relatively small number of participants. To generalize our data it could be important to perform approximately the same research with a greater number of participants and of different education levels. Finally, the situation in Belgium on the legalization of e-cigs containing e-liquid with nicotine is not yet clear. At the time of the research, e-liquid with nicotine could not be purchased in Belgium. When participants ran out of supply (we provided enough material for approximately two months) and wanted to continue using the e-cig with nicotine e-liquid, they were forced to buy it online. This could be one of the possible explanations for the decrease in quit rates. When people, ready to switch to an e-cig, are severely restricted in terms of accessibility of nicotine-containing e-liquids, the success of e-cigs may be endangered. For the e-cig to be and remain successful, it is important that people have easy access to nicotine containing e-liquids.

## 5. Conclusion

In a series of controlled lab sessions with e-cig-naïve tobacco smokers, second-generation e-cigs were shown to be immediately and highly effective in reducing abstinence-induced cigarette craving and withdrawal symptoms, while not resulting in increases in eCO. *Ad libitum* use of e-cigs—in between and until six months after the lab sessions—resulted in remarkable reductions in or (biologically confirmed) complete abstinence from tobacco smoking in almost half of the participants who had no intention to quit smoking. Eight months after the start of the study 21% of all participants were completely abstinent from tobacco cigarettes. Similar reduction/cessation rates were obtained with guided *versus* non-guided switching to e-cigs. Part of the observed efficacy of e-cigs in this study may be related to the fact that they allowed to maintain relatively high blood nicotine levels and showed an excellent experienced benefits/complaints ratio, especially in comparison with continued tobacco smoking. Larger randomized controlled trials in smokers wanting to quit and in clinical groups of people suffering from smoking-related disease are now needed to confirm and expand these encouraging observations.

## References

[1] World Health Organization (WHO) (2013). WHO Report on the Global Tobacco Epidemic: Enforcing Bans on Tobacco Advertising, Promotion and Sponsorship.

[2] GfK Significant Rookgedrag. in België.: Een. Rapport aan Stichting tegen Kanker. www.kanker.be/sites/default/files/rookenquete_2013.pdf.

[3] TNS Opinion & Social Attitudes of Europeans towards Tobacco. http://ec.europa.eu/public_opinion/archives/ebs/ebs_385_en.pdf.

[4] World Health Organization (WHO) (2009). Global Health Risks: Mortality and Burden of Disease Attributable to Selected Major Risks.

[5] World Health Organization (WHO) (2012). WHO Global Report: Mortality Attributable to Tobacco.

[6] Jha P., Peto R. (2014). Global effects of smoking, of quitting, and of taxing tobacco. New Engl. J. Med..

[7] Pirie K., Peto R., Reeves G.K., Green J., Beral V. (2013). The 21st century hazards of smoking and benefits of stopping: A prospective study of one million women in the UK. Lancet.

[8] Ng M., Freeman M.K., Fleming T.D., Robinson M., Dwyer-Lindgren L., Thomson B., Wollum A., Sanman E., Wulf S., Lopez A.D. (2014). Smoking prevalence and cigarette consumption in 187 countries, 1980–2012. J. Am. Med. Assoc..

[9] Wu P., Wilson K., Dimoulas P., Mills E.J. (2006). Effectiveness of smoking cessation therapies: A systematic review and meta-analysis. BMC Public Health.

[10] Cahill K., Stevens S., Lancaster T. (2014). Pharmacological treatments for smoking cessation. J. Am. Med. Assoc..

[11] Etter J.-F., Stapleton J.A. (2006). Nicotine replacement therapy for long-term smoking cessation: A meta-analysis. Tob. Control.

[12] Dhelaria R.K., Friderici J., Wu K., Gupta E., Khan C., Rothberg M.B. (2012). Effectiveness of varenicline for smoking cessation at 2 urban academic health centers. Eur. J. Intern. Med..

[13] Pierce J.P., Cummins S.E., White M.M., Humphrey A., Messer K. (2012). Quitlines and nicotine replacement for smoking cessation: Do we need to change policy?. Annu. Rev. Public Health.

[14] Dawkins L. (2013). Why is it so hard to quit smoking?. Psychologist.

[15] Rose J.E., Salley A., Behm F.M., Bates J.E., Westman E.C. (2010). Reinforcing effects of nicotine and non-nicotine components of cigarette smoke. Psychopharmacology.

[16] Russell M.A. (1974). The smoking habit and its classification. Practitioner.

[17] Russell M.A. (1976). Low-tar medium-nicotine cigarettes: A new approach to safer smoking. Br. Med. J..

[18] McNeill A., Munafo M.R. (2013). Reducing harm from tobacco use. J. Psychopharmacol..

[19] Harm Reduction: Concepts and Practices. www.tobaccoharmreduction.org/faq/harmreduction.htm.

[20] Rodu B. (2011). The scientific foundation for tobacco harm reduction, 2006–2011. Harm Reduct. J..

[21] Rodu B., Godshall W.T. (2006). Tobacco harm reduction: An alternative cessation strategy for inveterate smokers. Harm Reduct. J..

[22] Cahn Z., Siegel M. (2010). Electronic cigarettes as a harm reduction strategy for tobacco control: A step forward or a repeat of past mistakes?. J. Public Health Policy.

[23] Polosa R., Rodu B., Caponnetto P., Maglia M., Raciti C. (2013). A fresh look at tobacco harm reduction: The case for the electronic cigarette. Harm Reduct. J..

[24] Burstyn I. (2014). Peering through the mist: Systematic review of what the chemistry of contaminants in electronic cigarettes tells us about health risks. BMC Public Health.

[25] Farsalinos K.E., Polosa R. (2014). Safety evaluation and risk assessment of electronic cigarettes as tobacco cigarette substitutes: A systematic review. Ther. Adv. Drug Saf..

[26] Hajek P., Etter J-F., Benowitz N., Eissenberg T., McRobbie H. (2014). Electronic cigarettes: Review of use, content, safety, effects on smokers and potential for harm and benefit. Addiction.

[27] Etter J.-F. (2013). The Electronic Cigarette: An. Alternative to Tobacco?.

[28] Etter J.-F. (2010). Electronic cigarettes: A survey of users. BMC Public Health.

[29] Goniewicz M.L., Lingas E.O., Hajek P. (2013). Patterns of electronic cigarette use and user beliefs about their safety and benefits: An internet survey. Drug Alcohol Rev..

[30] Farsalinos K.E., Spyrou A., Tsimopoulou K., Stefopoulos C., Romagna G., Voudris V. (2014). Nicotine absorption from electronic cigarette use: Comparison between first and new-generation devices. Sci. Rep..

[31] Farsalinos K.E., Romagna G., Tsiapras D., Kyrzopoulos S., Voudris V. (2014). Characteristics, perceived side effects and benefits of electronic cigarette use: A worldwide survey of more than 19,000 consumers. Int. J. Environ. Res. Public Health.

[32] Joyetech. www.joyetech.com/product/eGoC.php.

[33] E-cig4U. www.e-cig4u.nl/Kanger-T2-CC.

[34] E-cig4U. www.e-cig4u.nl/dk-liquid-pg-vg.

[35] Joyetech eGo-C. Picture by Courtesy of E-cig4U. http://www.e-cig4u.nl/.

[36] Kanger T2 CC. Picture by Courtesy of E-cig4U. http://www.e-cig4u.nl/.

[37] Urbaniak G.C., Plous S. Research Randomizer (Version 4.0) (Computer software). www.randomizer.org/.

[38] Salimetrics (2009–2012). Salivary Assay Kits. www.salimetrics.com/salivary-assay-kits.

[39] Bedfont Scientific Ltd. piCO+ Smokerlyzer^®^: Operating Manual. www.bedfont.com/downloads/pico+/piCO+_English_v2_iss10.pdf.

[40] Fagerström K. (2012). Determinants of tobacco use and renaming the FTND to the Fagerström test for cigarette dependence. Nicotine Tob. Res..

[41] Beck A.T., Steer R.A., Brown G.K. (1996). Beck Depression Inventory.

[42] Singleton E.G., Anderson L.M., Heishman S.J. (2003). Reliability and validity of the tobacco craving questionnaire and validation of a craving-induction procedure using multiple measures of craving and mood. Addiction.

[43] Toll B.A., O’Malley S.S., McKee S.A., Salovey P., Krishnan-Sarin S. (2007). Confirmatory factor analysis of the Minnesota Nicotine Withdrawal Scale. Psychol. Addict. Behav..

[44] Dockrell M., Morison R., Bauld L., McNeill A. (2013). E-cigarettes: Prevalence and attitudes in Great Britain. Nicotine Tob. Res..

[45] Caponnetto P., Auditore R., Russo C., Cappello G.C., Polosa R. (2013). Impact of an electronic cigarette on smoking reduction and cessation in schizophrenic smokers: A prospective 12-month pilot study. Int. J. Environ. Res. Public Health.

[46] Caponnetto P., Campagna D., Cibella F., Morjaria J.B., Caruso M., Russo C., Polosa R. (2013). Efficiency and safety of an electronic cigarette (ECLAT) as tobacco cigarettes substitute: A prospective 12-month randomized control design study. PLoS ONE.

[47] Polosa R., Caponnetto P., Morjaria J.B., Papale G., Campagna D., Russo C. (2011). Effect of an electronic nicotine delivery device (e-Cigarette) on smoking reduction and cessation: A prospective 6-month pilot study. BMC Public Health.

[48] Polosa R., Morjaria J.B., Caponnetto P., Campagna D., Russo C., Alamo A., Amaradio M., Fisichella A. (2014). Effectiveness and tolerability of electronic cigarette in real-life: A 24-month prospective observational study. Intern. Emerg. Med..

[49] Dawkins L., Turner J., Roberts A., Soar K. (2013). ‘Vaping’ profiles and preferences: An online survey of electronic cigarette users. Addiction.

[50] Etter J.-F., Bullen C. (2014). A longitudinal study of electronic cigarette users. Addict. Behav..

[51] Bullen C., McRobbie H., Thornley S., Glover M., Lin R., Laugesen M. (2010). Effect of an electronic nicotine delivery device (e cigarette) on desire to smoke and withdrawal, user preferences and nicotine delivery: Randomised cross-over trial. Tob. Control.

[52] Dawkins L., Corcoran O. (2013). Acute electronic cigarette use: Nicotine delivery and subjective effects in regular users. Psychopharmacology.

[53] Dawkins L., Turner J., Hasna S., Soar K. (2012). The electronic-cigarette: Effects on desire to smoke, withdrawal symptoms and cognition. Addict. Behav..

[54] Vansickel A.R., Cobb C.O., Weaver M.F., Eissenberg T.E. (2010). A clinical laboratory model for evaluating the acute effects of electronic “cigarettes”: Nicotine delivery profile and cardiovascular and subjective effects. Cancer Epidemiol. Biomark. Prev..

[55] Vansickel A.R., Weaver M.F., Eissenberg T. (2012). Clinical laboratory assessment of the abuse liability of an electronic cigarette. Addiction.

[56] McQueen A., Tower S., Sumner W. (2011). Interviews with “vapers”: Implications for future research with electronic cigarettes. Nicotine Tob. Res..

[57] Dawkins L., Turner J., Crowe E. (2012). Nicotine derived from the electronic cigarette improves time-based prospective memory in abstinent smokers. Psychopharmacology.

[58] Kralikova E., Novak J., West O., Kmetova A., Hajek P. (2013). Do e-cigarettes have the potential to compete with conventional cigarettes? A survey of conventional cigarette smokers’ experiences with e-cigarettes. Chest.

[59] Siegel M.B., Tanwar K.L., Wood K.S. (2011). Electronic cigarettes as a smoking-cessation tool: Results from an online survey. Am. J. Prev. Med..

[60] Wagener T.L., Meier E., Hale J.J., Oliver E.R., Warner M.L., Driskill L.M., Gillaspy S.R., Siegel M.B., Foster S. (2014). Pilot investigation of changes in readiness and confidence to quit smoking after e-cigarette experimentation and 1 week of use. Nicotine Tob. Res..

[61] Caponnetto P., Polosa R., Russo C., Leotta C., Campagna D. (2011). Successful smoking cessation with electronic cigarettes in smokers with a documented history of recurring relapses: A case series. J. Med. Case Rep..

[62] Farsalinos K.E., Romagna G. (2013). Chronic idiopathic neutrophilia in a smoker, relieved after smoking cessation with the use of electronic cigarette: A case report. Clin. Med. Insights Case Rep..

[63] Bullen C., Howe C., Laugesen M., McRobbie H., Parag V., Williman J., Walker N. (2013). Electronic cigarettes for smoking cessation: A randomised controlled trial. The Lancet.

[64] Bullen C., Williman J., Howe C., Laugesen M., McRobbie H., Parag V., Walker N. (2013). Study protocol for a randomised controlled trial of electronic cigarettes *versus* nicotine patch for smoking cessation. BMC Public Health.

[65] Etter J.-F., Bullen C. (2011). Electronic cigarette: Users profile, utilization, satisfaction and perceived efficacy. Addiction.

[66] Van Staden S.R., Groenewald M., Engelbrecht R., Becker P.J., Hazelhurst L.T. (2013). Carboxyhaemoglobin levels, health and lifestyle perceptions in smokers converting from tobacco cigarettes to electronic cigarettes. South Afr. Med. J..

[67] Etter J.-F., Bullen C. (2011). Letters: Saliva cotinine levels in users of electronic cigarettes. Eur. Respir. J..

[68] Vansickel A.R., Eissenberg T. (2012). Electronic cigarettes: Effective nicotine delivery after acute administration. Nicotine Tob. Res..

[69] Etter J.-F. (2014). Levels of saliva cotinine in electronic cigarette users. Addiction.

[70] Barbeau A.M., Burda J., Siegel M. (2013). Perceived efficacy of e-cigarettes *versus* nicotine replacement therapy among successful e-cigarette users: A qualitative approach. Addiction Sci. Clin. Pract..

